# Superior mesenteric artery syndrome leading to reversible mucosal gangrene

**DOI:** 10.1002/ams2.283

**Published:** 2017-05-08

**Authors:** Satomi Uemura, Kei Suzuki, Naoyuki Katayama, Hiroshi Imai

**Affiliations:** ^1^ The Emergency and Critical Care Center Mie University Hospital Tsu Mie Japan; ^2^ Department of Hematology and Oncology Mie University Graduate School of Medicine Tsu Mie Japan

## Abstract

We describe a case in which gastrointestinal distention due to superior mesenteric artery syndrome (SMAS) developed into membranous gangrene, which in turn led to septic shock in a 60‐year‐old woman with cerebral palsy and cachexia. The association with SMAS and septic shock is considered extremely rare, it is important to consider this combination especially in cachectic patients with gastric distension accompanying refractory shock unknown etiology.

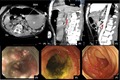

Dear Editor,

Superior mesenteric artery (SMA) syndrome (SMAS) is a rare cause of proximal bowel obstruction resulting from compression of the duodenum secondary to narrowing of the space between the aorta and SMA. Here, we report a case of reversible acute mucosal gangrene (MG) accompanying septic shock that resulted from SMAS.

A 60‐year‐old woman, bedridden because of cerebral palsy and admitted at a nursing‐care facility a week before presentation, was brought to the emergency department with multiple vomiting episodes. She received normal solid nutriments, but became recumbent after admission to the facility. On arrival, she was in shock with a systolic blood pressure of approximately 100 mmHg and heart rate of 150 b.p.m. She was cachectic (BMI: 16.8 kg/m^2^) and her abdomen was distended. Plain computed tomography (CT) showed hepatic portal venous gas accompanying gastric and duodenal distention with abrupt narrowing at the third part of the duodenum, with the SMA crossing anterior to the transition point (Fig. 1A). Additionally, peripherally distributed air in the intrahepatic portal venous system within 2 cm of the liver surface was found but no intestinal pneumatosis was noted, except in the duodenum. Reconstructed CT showing a reduced aortomesenteric angle (AMA) (17°; Fig. 1B) suggested SMAS.^1^, ^2^ Laboratory examination revealed metabolic acidosis (pH 7.3; lactate, 12.1 mmol/L; ${\text{HCO}}_{3}^{-}$, 7.9 mmol/L; base excess, −15.6 mmol/L). Contrast‐enhanced CT after stomach decompression by nasogastric tube indicated the expanding of AMA of 42°; no evidence of bowel necrosis or occlusion of the mesenteric arteries was observed (Fig. 1C). Upper endoscopy also confirmed superficial MG (Fig. 1D). The foul smelling drainage fluid from the nasogastric tube suggested fluid retention lasting several days. Despite sufficient fluid resuscitation (i.v. extracellular fluid of 2000 mL), severe hypotension developed with poor lactate clearance. Additional blood investigation revealed an extremely high serum procalcitonin level (>75 ng/mL; normal, <0.10 ng/mL). Although blood culture did not yield a causative pathogen, septic shock^3^ with bacterial translocation through gangrenous membrane resulting from increased intraduodenal pressure due to SMAS was considered.^4^ Because vascular occlusion and transmural bowel necrosis were both excluded by radiology and/or endoscopy, AMA was improved by decompression with nasogastric drainage. The patient received antibiotics (meropenem, 1 g every 12 h, which was adjusted to her renal function) with vasopressors and she recovered from shock in approximately 48 h. Meropenem was discontinued in 14 days with improvement of her general condition and serum procalcitonin level (0.40 ng/mL).^5^ Repeated upper endoscopy also revealed gradual improvement of the MG (Fig. 1E, F).

**Figure 1:**
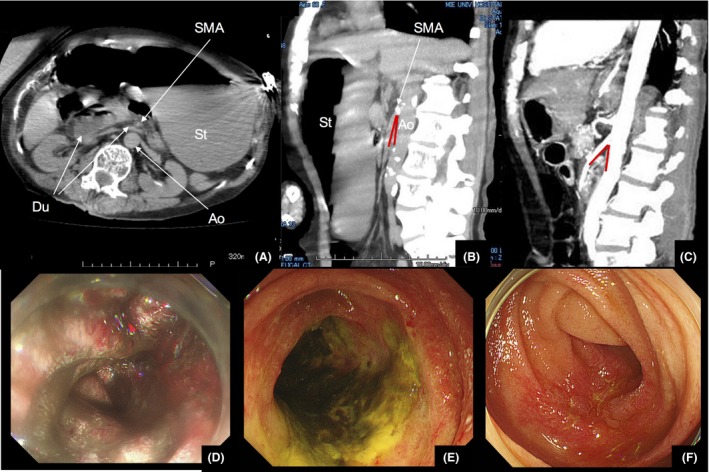
Computed tomography (CT) and endoscopic images of a 60‐year‐old woman with reversible acute mucosal gangrene accompanying septic shock that resulted from superior mesenteric artery (SMA) syndrome. A, CT axial section showing compression of the third part of the duodenum between the SMA and abdominal aorta (Ao), with proximal duodenal and gastric dilatation. B, Sagittal multiplanar reconstruction contrast‐enhanced CT showing the SMA arising from the aorta and forming a narrow aortomesenteric angle (AMA) (red lines) of 17° (in normal condition, 45–60°). C, Contrast CT carried out after gastric decompression showed the improvement of AMA (red lines) at 42° and revealed no mesenteric artery occlusion or mucosal ischemia. D, Emergency upper endoscopy suggested acute ischemia that resulted in superficial membranous gangrene; however, there was no evidence of transmural necrosis. E, F, Repeated upper endoscopy showed gradual improvement of the mucosal gangrene. Images in panels (E) and (F) were obtained on day 10 and 21, respectively. Du, duodenum; St, stomach.

This case reveals two important points: (i) gastrointestinal distention due to SMAS occasionally causes MG, leading to septic shock, (ii) although surgery should be considered, superficial MG induced by SMAS gradually improved with conservative therapy in this case without vascular occlusion or mesenteric ischemia.

Significant weight loss is one of the main risk factors associated with SMAS and prolonged immobility contributes to SMAS development by AMA narrowing, with dilation of the stomach in the present case. Duodenum microbiota has a low potential to cause bacterial translocation compared with intestinal translocation; however, the retained gastroduodenal contents may have contributed to the septic shock development in this case. The association between SMAS and septic shock is rare and it is important to consider this, especially in cachectic patients with gastric distension accompanying refractory shock with unknown etiology.

## Conflict of interest

None declared. The protocol for this study has been approved by a suitably constituted Ethics Committee of the institution and conforms to the provisions of the Declaration of Helsinki. Informed consent was obtained from the guardian.
